# Fabrication of Capacitive Acoustic Resonators Combining 3D Printing and 2D Inkjet Printing Techniques

**DOI:** 10.3390/s151026018

**Published:** 2015-10-14

**Authors:** Rubaiyet Iftekharul Haque, Erick Ogam, Christophe Loussert, Patrick Benaben, Xavier Boddaert

**Affiliations:** 1 Centre Microélectronique de Provence (CMP), École Nationale Supérieure des Mines de Saint-Étienne, 13541 Gardanne, France; E-Mail: benaben@emse.fr; 2 Laboratoire de Mécanique et D’Acoustique UPR7051 CNRS, 31 Chemin Josep Aiguier, 13402 Marseille, France; E-Mail: ogam@lma.cnrs-mrs.fr; 3 TAGSYS RFID, 13600 La Ciotat, France; E-Mail: christophe.loussert@tagsysrfid.com

**Keywords:** acoustic transducer, resonator, printed electronics, inkjet printing, 3D printing

## Abstract

A capacitive acoustic resonator developed by combining three-dimensional (3D) printing and two-dimensional (2D) printed electronics technique is described. During this work, a patterned bottom structure with rigid backplate and cavity is fabricated directly by a 3D printing method, and then a direct write inkjet printing technique has been employed to print a silver conductive layer. A novel approach has been used to fabricate a diaphragm for the acoustic sensor as well, where the conductive layer is inkjet-printed on a pre-stressed thin organic film. After assembly, the resulting structure contains an electrically conductive diaphragm positioned at a distance from a fixed bottom electrode separated by a spacer. Measurements confirm that the transducer acts as capacitor. The deflection of the diaphragm in response to the incident acoustic single was observed by a laser Doppler vibrometer and the corresponding change of capacitance has been calculated, which is then compared with the numerical result. Observation confirms that the device performs as a resonator and provides adequate sensitivity and selectivity at its resonance frequency.

## 1. Introduction

A capacitive acoustic transducer is an electromechanical acoustic system that usually consists of a fixed backplate electrode and a flexible diaphragm separated by an air gap to form a parallel plate capacitor [1]. The incident acoustic pressure leads to the deflection of the diaphragm, thereby providing a capacitance variation in response to the change in air gap. A capacitive acoustic transducer generally suffers from over-damping, as a thin layer of air is trapped in between the electrodes. To overcome this issue, capacitive acoustic transducers are usually designed and fabricated with porous membranes or/and perforated backplates [1].

There are different types of configurations available to fabricate capacitive acoustic devices. For example, the counterelectrode can be located beneath the diaphragm and have a small number of holes, or the counterelectrode can be perforated with a large number of holes and be located above the diaphragm. In addition to that, there is dual-backplate microphone that has two backplates, one on each side of the diaphragm, or with dual-membranes [1]. The design of acoustic sensors can vary based on the application requirements and fabrication process.

**Figure 1:**
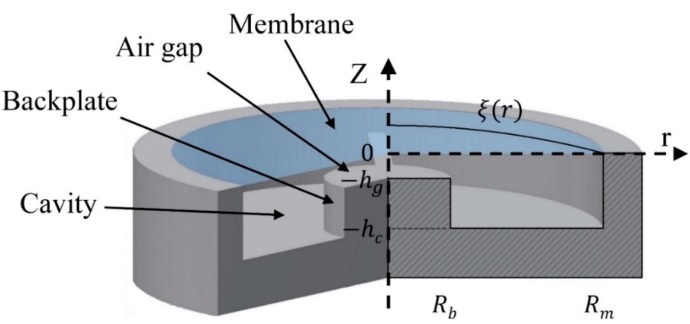
Schematic diagram of the capacitive acoustic transducer.

In recent times, to fulfill the industrial requirement of a cost efficient printed capacitive acoustic sensor having the proper sensitivity and selectivity to develop highly integrated RFID solutions [2], a new design to fabricate capacitive acoustic transducer has been proposed (Figure 1) [3,4], which can also be used to fabricate capacitive acoustic resonant sensors with adequate selectivity and selectivity, as described by Haque *et al.* [5].

Different materials have been used to fabricate acoustic transducers depending on their types [1]. In terms of electrical properties, metals are appropriate for diaphragm and backplate applications. However, metals are vulnerable to the aggressive thermal and chemical environment employed during the device manufacturing process, as well as less resistant to corrosion during its lifetime. Therefore to overcome these problems, most microelectromechanical systems (MEMS) sensors and actuators are fabricated on silicon substrates using conventional subtractive methods, such as photolithographic patterning and surface micromachining, *etc.* Occasionally, organic thin films with desired mechanical properties that are chemically more robust have been used as support membranes to fabricate diaphragms for the transducers as well [6].

Although subtractive methods, like photolithographic patterning, are well-established techniques to fabricate acoustic transducers, they require various depositions, masking and etching processes to develop a 3D structure. Moreover, these techniques exhibit higher production cost and process times and generate large volumes of hazardous waste that damage the environment. On the other hand, vacuum deposition techniques for electronic devices fabrication are energy intensive and provide low resolution [7]. In addition, the chemical vapor deposition technique, which is also widely used to fabricate MEMS devices [8,9,10,11], requires the use of volatile precursors and elevated temperatures for the deposition of the layer and can be quite expensive [12]. Thus, to reduce material losses and to be more cost efficient, the search for a new technique led us to an innovative method—printing techniques—which has been employed to fabricate acoustic transducers during this work.

The use of revolutionary printing techniques to develop electronic devices provides new opportunities for the microelectronics industries. Light weight and increased robustness, as the components are flexible, are the main advantages of printed electronics [13]; as a result they withstand heat, rough handling and harsh environmental conditions better than conventional electronics [14,15,16]. Furthermore, printed electronics techniques can be used alone or in combination with conventional microelectronic components, such as silicon chips for a range of different applications as well [17]. Amongst the well-developed printing techniques, inkjet printing is a contact-free additive printing technique for positioning droplets of liquid material with high precision onto a substrate. Inkjet printing operates at room temperature under ambient conditions and involves the use of fewer hazardous chemicals. The shift from high vacuum, high purity manufacturing to printing processes reduces the product manufacturing cost. Moreover, this technique is flexible, versatile and can be set up with relatively low effort, as no masks or screens are needed [7,18,19]. Therefore, the inkjet printing technique could be a potential alternative for microelectronics fabrication because of its high precision printing ability, cost efficiency and less waste production. The curing of the deposited ink in order to remove solvents, to initiate the film cohesion and to improve the functionality of the printed layer, can be carried out using either conventional thermal heat sintering or other selective techniques such as laser, microwave, joule heating [20,21,22] and photonic sintering technique [7,23,24].

Inkjet printing can be used to fabricate different types of structures, such as electrical interconnects; embedded electrical passive structures, such as conductors and resistors, organic electronics, displays, and even to develop under-bump metallization [25]. Inkjet printing technology can also be used to develop 3D structures, such as microelectromechanical systems (MEMS) and other structures. Fuller *et al.* have shown that 3D metallic structures can be formed by colloid ink containing nanoparticles [26]. In addition, 3D structures can also be formed by printing several layers of microstructures and sacrificial layers. This technique can be used to fabricate a wide variety of microstructures, including different forms of MEMs structures that consist of voids or cavities [27], where the void or cavity is formed after removal of the sacrificial layer by a subtractive process. However, to form devices with large cavities or voids on a millimeter scale with complex geometry is difficult using this technique. These issues can be addressed by fabricating 3D structures with large cavities or voids, such as used in MEMS acoustic transducer, using emerging 3D printing methods directly.

The 3D printing method is a powerful additive manufacturing method, which can be used to fabricate complex structures directly without the requirement of any etching or micromachining [28]. This technique allows the simultaneous printing of multiple materials, having various properties [29,30,31] in the same structure without the requirement for any post-curing.

It appears that the prior art has not led to the fabrication of acoustic transducers combining printed electronics, direct write inkjet techniques and emerging three-dimensional printing methods to fabricate 3D electronics devices. The main goal of this work is to develop a prototype of a capacitive acoustic transducer using printing techniques with appropriate sensitivity and selectivity, where 3D printing and 2D inkjet printing methods are combined. In this regard, during this work we developed a new process, where patterned 3D structures for MEMS devices were built directly by a 3D printing method and used as a substrate to print electrically conductive ink layer by a 2D inkjet printing technique. In addition, the printing process is also developed by printing conductive layers on thin films to fabricate membranes with applied tension along the periphery.

## 2. Acoustic Transducer Design

The schematic of the design of the capacitive acoustic transducer is presented in Figure 1. It consists of a central cylindrical rigid backing electrode of small radius surrounded by a flat annular cavity below a vibrating membrane clamped at its periphery. During this work, the possibility of developing an acoustic resonator using printing techniques which will fulfill the requirements of a nominal capacitance value of around 0.5 to 3 pF, which should provide a capacitance variation of about 1 to 10 fF for 80 dB_SPL_ incident acoustic pressure have been investigated. In addition, the sensor should have selectivity with a Q-factor above 25 at its first resonance frequency. Haque *et al.* [5] have presented the optimum range of parameters for the design that will satisfy the device requirements. According to the study, the best optimum set of parameters are a membrane radius of 8.1 mm, backplate radius of 871 µm, cavity height of around 3987 µm and air gap of around 80 µm and membrane thickness of 19.8 µm along with a membrane tension of 2158 N/m.

The capacitance variation (∆*C*) of the capacitive transducer can be expressed as:

(1) $$\text{Δ}C=C_{i}\frac{\left|{\langle \text{ξ}\rangle }_{Se}\right|}{h_{g}}$$

where *C_i_* represents the static capacitance of the transducer, *h_g_* is the air gap under an applied external DC potential and <ξ>*_Se_* represents the average small signal deformation of the effective membrane. For details see [5].

The selectivity of the acoustic resonator generally depends on its natural frequency or resonance frequency, and the Q-factor (*Q_f_*), which is related to the energy loss of the vibrating diaphragm [32], is characterized by a resonator’s bandwidth relative to its center frequency. Generally, in frequency domain, *Q*-factor is expressed as:

(2) $$Q_{f}=\frac{f_{r}}{\text{Δ}f}$$

where *f_r_* is the resonant frequency and ∆*f* is the half-power bandwidth, also known as the bandwidth over which the power of the vibration is greater than half the power at the resonant frequency. A high *Q_f_* value indicates a low rate of energy loss relative to the stored energy of the resonator [33,34]. Therefore, in the case of acoustic resonator a higher *Q*-factor is desirable to achieve higher selectivity.

## 3. Experimental Details

### 3.1. Materials and Printing Systems

Three dimensional (3D) printing was performed using MED610 polyjet ink from Stratasys (Eden Prairie, MN, USA), where the thickness of each printed layer is around 16 µm. Membrane fabrication was carried out using thin polyethylene terephthalate (PET) film or Mylar film with thickness of 23 μm purchased from Technifilm Company (Valence, France). Prior to printing the conductive layer, the surface of these substrates was rinsed with isopropanol and dried using blown nitrogen. Two different silver nanoparticle based inks, namely Silverjet ANP DGP-HR ink having 35–40 wt % of metal content from Advanced Nano Products (ANP, Sejong-si, Souh Korea), and Suntronics U5714 from SunChemical (Parsippany, New Jersey, USA) having 40 wt % of metal content have been used to print electrodes on 3D printed structures and on thin polymer film, respectively. Three dimensional (3D) printing was performed using an Objet EDEN 260 V polyjet 3D printing system from Stratasys (Eden Prairie, MN, USA) and direct write 2D inkjet printing of conductive silver layer was performed by a Dimatix DMP-2800 material inkjet printing system from Fujifilm (Santa Clara, CA, USA) using 10 pL cartridges consisting of 16 nozzles.

### 3.2. Device Fabrication

**Figure 2:**
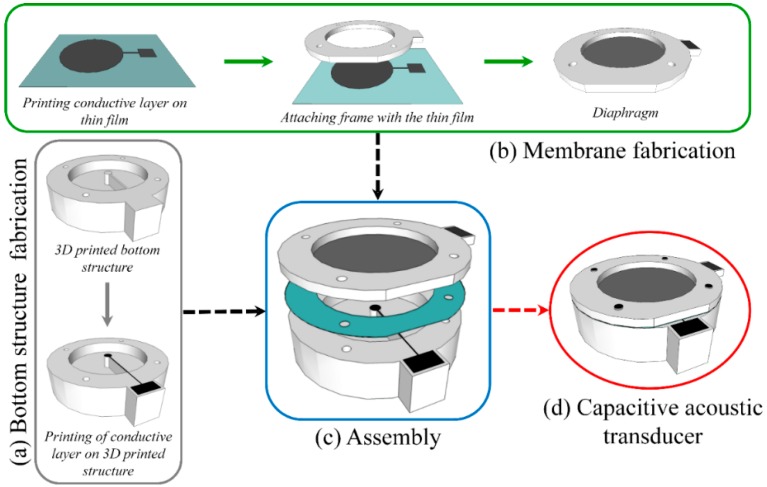
Fabrication steps of acoustic sensor combining 2D inkjet printing and 3D printing techniques (**a**) bottom structure fabrication; (**b**) membrane fabrication; (**c**) assembly and (**d**) capacitive acoustic sensor after assembly.

This work demonstrates an alternative method to fabricate an acoustic transducer, where the 3D structure of a capacitive acoustic transducer with a rigid backplate of small radius and a flat annular cavity has been fabricated using a 3D printing method without any etching or micromachining steps. Then a conductive silver layer has been inkjet printed on the 3D printed structure, based on the technique developed by Haque *et al.* [35], and sintered (Figure 2a).

For the fabrication of the diaphragm, initially a thin organic film was mounted on a specifically designed film holder and the required tension has been applied at the circumference of the film. Finally, the electrically conductive silver ink layer is inkjet-printed on the pre-stressed thin organic film and sintered to obtain a conductive layer. A rigid frame made by a 3D printing method has been glued to the thin organic film on the opposite surface of the printed conductive layer using adhesive. The membrane with printed conductive layer attached on the rigid frame has then been cut-off from the film holder, as illustrated in Figure 2b. The detailed schematics of diaphragm fabrication can be seen in Figure S1 (Supplementary Materials).

**Figure 3:**
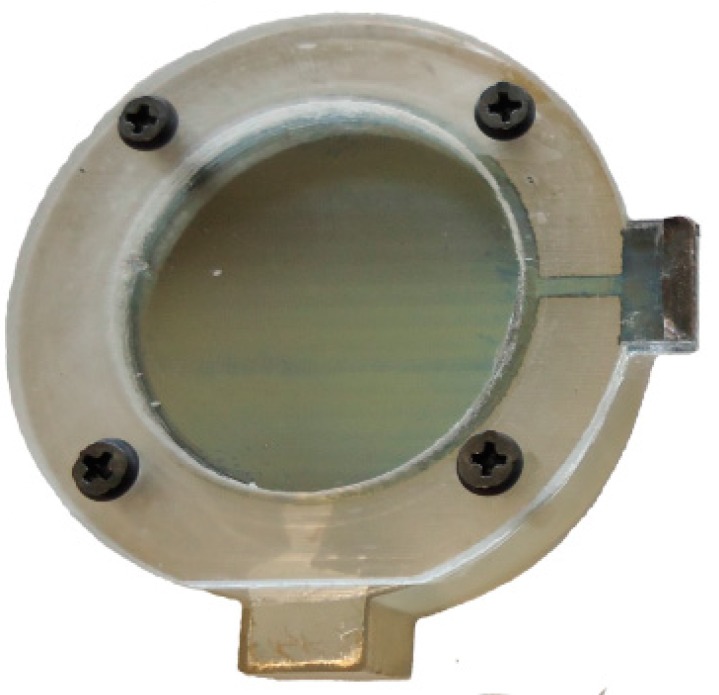
Printed capacitive acoustic transducer.

Thereafter, the bottom structure of the transducer and the membrane have been joined together via screws, separated by a 3D printed spacer in a way so that the printed conductive layers were positioned facing each other. Figure 2 illustrates the complete fabrication steps and Figure 3 presents the prototype of the printed capacitive acoustic sensor.

### 3.3. Experimental Procedure

After 3D printing of the structure, the polishing was performed using a LaboPol-5 grinding and polishing machine from Struers (Ballerup, Denmark) from 50 to 500 rpm where abrasive grinding papers and dispersed alumina nanoparticle having different particle sizes were used. Roughness of the 3D printed structure before and after polishing are investigated using an optical profilometer (model: WYKO NT110 from Veeco, Plainview, NY, USA), where the scanned surface area was 1.2 × 0.9 mm^2^. The surface energy of the polished 3D printed substrate was also measured. In this regard, the contact angle method has been carried out using an OCA200 goniometer from Dataphysics (Filderstadt, Germany). Three different solvents, namely H_2_O, diiodomethane and 40% ethylene glycol were used. Thereafter, the Owens-Wendt-Rabel-Kälbe (OWRK) method [36,37,38] was used to calculate the surface energy. Next, the conductive layer is printed on the 3D printed structure with a drop spacing of 30 µm, which is followed by drying and sintering steps. Experiments show that to obtain a conductive layer, sintering of the ANP silver nanoparticle-based ink needs to be performed at temperatures between 120 to 250 °C for a sufficient time [39]. As the 3D printed structures have very low heat deflection temperature (*T_HD_*) of 45–50 °C and glass transition temperature (*T_g_*) of 52–54 °C [28,40,41], the sintering step of the conductive layer using conventional thermal heat is not possible. The sintering of the 3D printed substrate in a conventional thermal heat oven causes structural damage. Therefore, during this work, sintering of the conductive layer on 3D printed structure has been performed by a photonic sintering technique [23,24,42] using a PulseForge 3200 (Novacentrix, Austin, TX, USA) equipped with a N20-VXI lamp. The maximum energy input that can be used during photonic sintering is mainly limited by the effective heat dissipation, which depends on the absorption coefficient, thermal conductivity, heat capacity and also the thickness of the substrate. Therefore, prior to the device fabrication step, the photonic sintering process has been optimized. In this regard, samples are prepared by printing silver-squares having an area of 1 cm^2^ on 3D printed substrate. After drying the as-printed layers at 30 °C under vacuum for 15 min, sintering of the samples were performed using photonic curing technique under different conditions, where the photonic sintering parameters, such as voltage (*V_B_*), pulse duration (*T_on_*), time between two pulses (*T_off_*) and number of pulses (*N_p_*) that corresponds to the exposed energy in J/cm^2^ are varied. Similarly for membrane fabrication, the inkjet printing of a conductive silver layer on thin polymer film was performed with a drop spacing of 25 µm on Mylar film. The conductive layers were then sintered using a conventional thermal heat oven at different sintering temperatures below the glass transition temperature (*T_g_*) of the substrate for different processing times.

The electrical properties of the printed conductive layer has been measured by a JANDEL (Linslade, UK) four point probing resistance measurement system (model: RM3-AR). The microstructure of the conductive layers were then studied by scanning electron microscopy (SEM) using an Ultra 55 instrument from Carl Zeiss (Oberkochen, Germany). The X-ray diffraction (XRD) of the printed layers were performed as well, using an X’pert Pro X-ray diffractometer from PANaltical (Almelo, The Netherlands), Cu-Kα X-rays of wavelength (λ) equals to 1.54059 Å for the 2θ range of 10° to 90° with the step size of 0.04°. In addition, the photonic sintered layers on the 3D printed substrates were also investigated by atomic force microscopy (AFM) using an Autoprobe CP II (Veeco, Plainview, NY, USA) in tapping mode.

**Figure 4:**
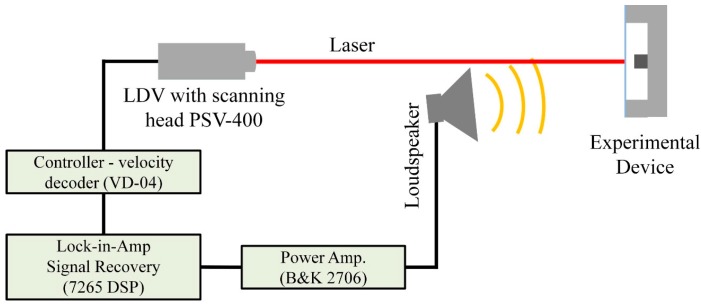
Schematic diagram of the experimental setup for the LDV measurement.

Once the sintering parameters of conductive layer on 3D printed substrate and on the thin film are optimized, the acoustic transducer was fabricated as described above. The measurement of the static capacitance of the device was conducted using a 4284A Precision LCR Meter (Agilent, Santa Clara, CA, USA). Dynamic characterization of the printed capacitive acoustic transducers have been performed using non-contact laser Doppler vibrometer (LDV) (Model: PSV-400 scanning laser vibrometer, Waldbronn, Germany) to understand their behavior under acoustic pressure harmonic excitation between 20 to 20 kHz. During this work, acoustic pressure excitations have been generated by a loudspeaker (B & C-DE-700-8, Bagno a Ripoli, Italy) and the corresponding response has been recorded using LDV in single-point mode and scanning mode. Figure 4 shows the schematic diagram of the experimental setup of the measurement using LDV (details of the experimental set up can be seen in Figure S2 in the Supplementary Materials section). In the case of single-point measurement mode, the laser spot is fixed at the center position of the membrane (to acquire the response spectrum); whereas during scanning measurement mode, the laser spot scan through the defined points (Figure S2c in Supplementary Materials section) on the surface of the membrane of the acoustic transducer (to acquire the surface deflection modes). Finally, the experimental observation is compared to the numerical result performed using COMSOL Multiphysics software. For details about numerical model building see [5].

## 4. Result and Discussion

### 4.1. Three-Dimensional (3D) Printed Structure

**Figure 5:**
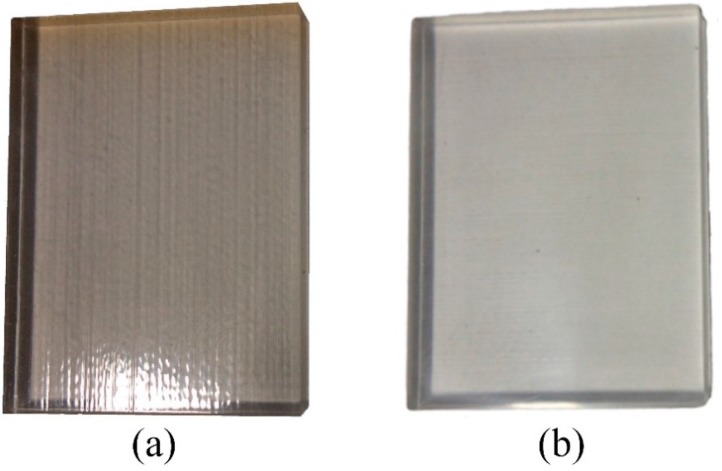
Three-dimensional (3D) printed substrate, (**a**) as printed; (**b**) after polishing.

Inkjet printing of a conductive layer requires a substrate with a smooth surface and appropriate surface energy, but 3D printed structures show relatively large surface roughness (Figure 5a). Therefore a polishing step is required before the printing process [35]. Figure 5b presents the 3D printed structure after polishing. The initial roughness of the as-printed 3D structure exhibits root-mean square roughness (*S_q_*) of 1450 ± 150 nm, which is reduced to 99.4 ± 17 nm after polishing. The roughness of the surface is correlated with the polishing time as well as the size of particles of the abrasive grinding paper and dispersed alumina nanoparticles.

It has been observed that the surface energy of the 3D printed substrate is 40.7 ± 0.7 mN/m, which is similar to that of the commercially available polyethylene terephthalate (PET) substrate having surface energy of 40.7 ± 0.2 mN/m. The low roughness and appropriate surface energy allows the inkjet printing of the conductive silver layer on the 3D printed substrates.

### 4.2. Characterization of Printed Layer on 3D Printed Substrate

Sheet resistance (*R_sq_*) of the printed silver layer on 3D printed substrate was measured using a four point probing resistance measurement system. Thereafter, resistivity (ρ) of the samples can be calculated using the following expression:

(3) $$\rho =R_{sq}.t$$

Here *t* represents the thickness of the inkjet-printed conductive silver layer, which is 1 ± 0.1 µm as measured using mechanical profilometer (model: XP-2, AMBiOS Technology, Santa Cruz, CA, USA). The electrical conductivity (σ) in S/m, which is reciprocal of the resistivity can then be calculated by Equation (4):

(4) $$\sigma =\frac{1}{\rho }=\frac{1}{R_{sq}.t}$$

**Figure 6:**
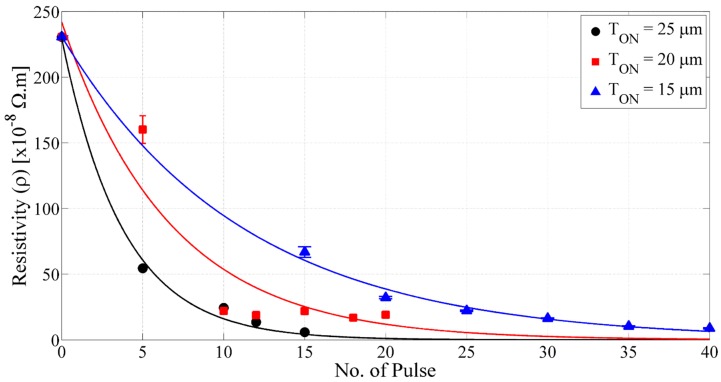
Resistivity of the inkjet-printed silver layer on 3D printed substrate (Dried and photonic sintered for constant *V_B_* = 400 V, *T_off_* = 1000 µs).

Figure 6 shows the resistivity of the conductive silver layers on 3D printed substrate. It has been observed that the conductivity increases with the increasing pulse duration.

Resistivity as low as 5.9 × 10^−8^ Ω·m on the 3D printed substrate was achieved for *V_B_* of 400 V, *T_on_* of 25 µs, *T_off_* of 1000 µs and *N_p_* of 15; however, the pilling-off of the layer was observed. Optimum sintering without any delamination of the conductive layer on the 3D printed substrate was achieved for *V_B_* of 400 V, *T_on_* of 15 µs, *T_off_* of 1000 µs and *N_p_* of 40, where the resistivity was 8.8 × 10^−8^ Ω·m.

**Figure 7:**
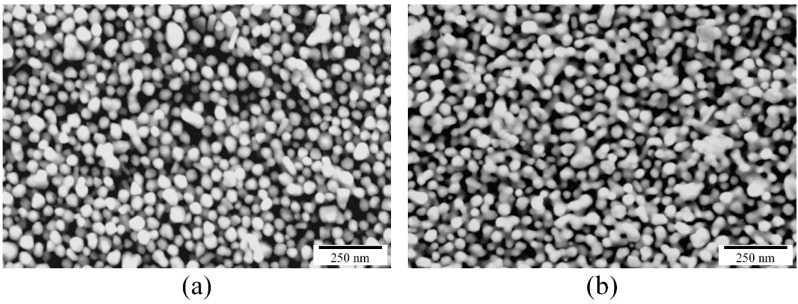
Microstructural evolution of the printed layer on 3D printed substrate, (**a**) dried (at 30 °C under vacuum for 10 min); and (**b**) photonic sintered after drying step (at *V_B_* = 400 V, *T_on_* = 15 µs, *T_off_* = 1000 µs, *N_p_* = 40).

The increase in electrical conductivity can be explained by the microstructural evolution due to the sintering of the nanoparticles, as illustrated in Figure 7a,b. The neck formation and the formation of larger particles take place due to the coalescence because of the photonic sintering of the silver nanoparticles.

**Figure 8:**
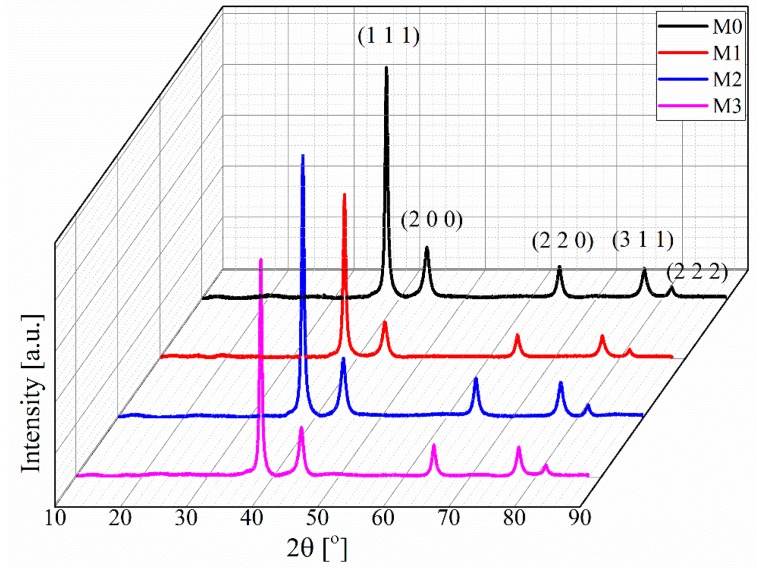
XRD pattern of the inkjet-printed printed silver nanoparticle on 3D printed substrates after photonic sintering.

The crystalline grain size of the inkjet-printed silver nanoparticles were determined using the Rietveld refinement technique of XRD patterns. Figure 8 illustrates the XRD patterns of the silver nanoparticles after photonic sintering on 3D printed substrates. A number of strong Bragg reflections can be seen. They correspond to the (111), (200), (220), (311) and (222) reflections of face centered cubic (fcc) silver.

During this study, the refinement of XRD patterns using the Rietveld method were performed by Maud software [43,44], and the obtained particle sizes are listed in Table 1. The particle size calculation from the XRD patterns indicates the increase of the grain size with the increasing number of pulse for constant values of *V_B_*, *T_on_* and *T_off_* on 3D printed substrate.

**Table 1:** Measured average particle size of the silver nanoparticles on 3D printed substrate from XRD patterns using Rietveld refinement technique.

Sample Code	Photonic Sintering Parameters				Average Grain Size *D*
	*V_B_*	*T_on_*	*T_off_*	*N_p_*	
	V	µs	µs		nm
M0	-	-	-	-	51.6 ± 1.3
M1	400	15	1000	15	58.6 ± 0.5
M2	400	15	1000	25	67.7 ± 2.5
M3	400	15	1000	40	87.2 ± 4.1

As the resistivity of the photonic sintered silver layer on the 3D printed substrate reduces as well with the increasing number of pulses for *V_B_*, *T_on_* and *T_off_* at constant values, therefore the reduction of electric resistivity can be linked to the grain size growth that has been observed by the Rietveld refinement of XRD analysis.

**Figure 9:**
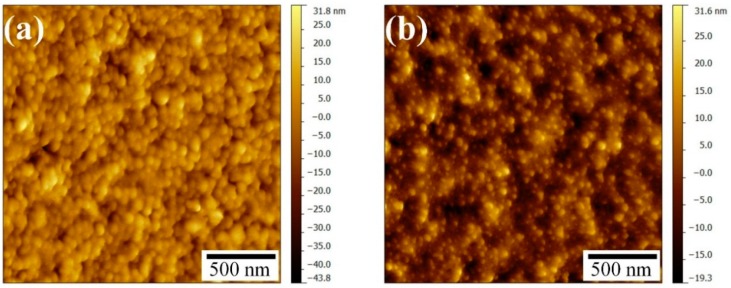
AFM micrograph of inkjet-printed conductive silver layer on the 3D printed substrate, (**a**) dried; (**b**) Photonic sintered after drying step (*V_B_* = 400 V, *T_on_* = 15 µs, *T_off_* = 1000 µs, *N_p_* = 40).

The roughness of the photonic sintered silver layer on 3D printed substrates was also measured from the AFM micrographs where the scanned surface area was 50 × 50 µm^2^, and are listed in Table 2. The average roughness of the silver layers was around 10 nm. Therefore, the photonic sintered printed silver layer provides a smooth surface finish on 3D printed substrates. Figure 9 presents AFM micrographs of the surface of the photonic sintered samples of silver conductive layers on the 3D printed substrate.

**Table 2:** Roughness of photonic sintered samples (for scanned surface area: 50 × 50 µm^2^).

Sample Code	Photonic Sintering Parameters				Roughness	
	*V_B_*	*T_on_*	*T_off_*	*N_p_*	*S_a_*	*S_q_*
	V	µs	µs		nm	nm
M0	-	-	-	-	7.1 ± 0.9	9.2 ± 1.3
M1	400	15	1000	15	6.4 ± 0.6	8.2 ± 0.7
M2	400	15	1000	25	7.5 ± 0.5	10.3 ± 1.3
M3	400	15	1000	40	6.0 ± 0.5	7.7 ± 0.7
*S_a_* → Arithmetic mean of roughness				*S_q_* → Root mean square of roughness		

Based on the tests on 3D printed substrates, the building blocks for the fabrication process of the capacitive acoustic transducer have been developed. The best photonic sintering result on the 3D printed substrate without delamination of the printed silver layer was obtained for the photonic sintering parameters of *V_B_* of 400 V, *T_on_* of 15 µs, *T_off_* of 1000 µs and *N_p_* of 40, which were used during the device fabrication step.

### 4.3. Conductive Layer Printed on Thin Film

**Figure 10:**
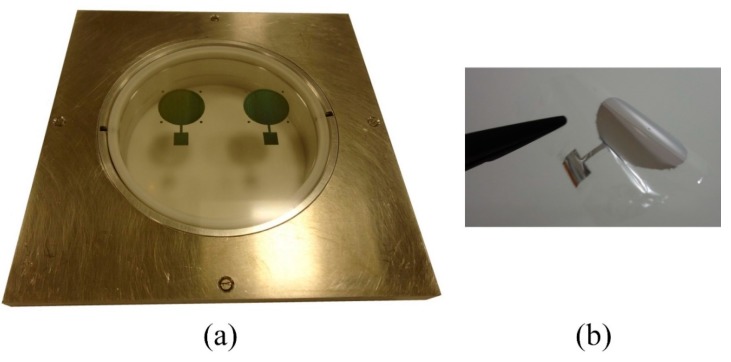
Inkjet printing on thin Mylar film, (**a**) mounted on thin film holder; (**b**) thin film after removal from the film holder.

During this work, a process was developed to fabricate a pre-stressed membrane by inkjet printing of a conductive silver layer on a thin Mylar film. As it is very difficult to work with very thin film due to static charging, a specific film mounting holder (Figure 10) has been designed to apply a tension to the thin film and hold it as a flat surface during the inkjet printing process.

**Figure 11:**
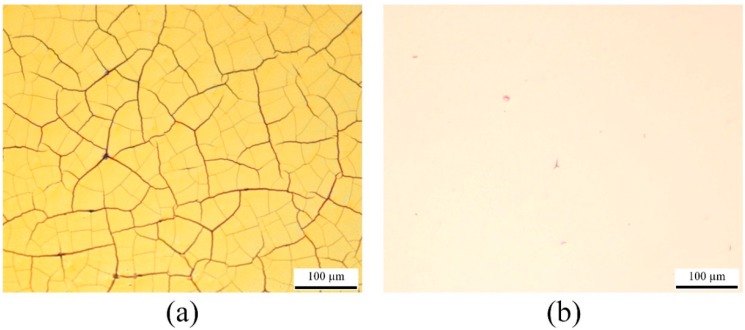
Optical microscopic image of the inkjet-printed conductive silver layer on thin polymer film sintered at 140 °C for 30 min, (**a**) tension applied after the printing and sintering of the layer; and (**b**) tension applied before the printing of the conductive layer.

It has been observed that the resistivity of the printed silver layer increases and eventually it becomes non-conductive, if the tension is applied at the periphery of the thin film after printing and sintering the conductive layer. This is caused by the formation of cracks on the conductive layer (Figure 11a). This problem can be solved by applying tension on the thin Mylar film before printing and sintering of the conductive layer, as shown in Figure 11b. However, in this case, occasional readjustment of the membrane tension is required as the tension is released during thermal heating.

Electrical measurements have showed that the conductivity of the printed silver layer having the average thickness of 500 ± 50 nm, increases with increasing sintering time. For example, the conductivity of the printed layer increases from 1.6 × 10^6^ S/m for sintering at 90 °C for 30 min to 5.5 × 10^6^ S/m for sintering at 140 °C for 30 min. The particle size of the nanoparticle for different sintering temperatures and times on thin Mylar films has been calculated by the Rietveld refinement technique using Maud software for the XRD pattern, which reveals that the average particle size increases with the increasing temperature and sintering time. The average conductivity and the gain size are listed in Table 3. During the device fabrication process, the inkjet-printed silver layer on pre-stressed thin polymer film was sintered at 140 °C for 30 min.

**Table 3:** Average conductivity and grain size of the inkjet-printed silver nanoparticles layers with different thermal sintering temperature and time.

Sintering Parameters		Conductivity	Grain Size
Temperature	Time		
°C	min	S·m^−1^	nm
90	30	1.6 × 10^6^	42.9 ± 0.8
120	30	3.3 × 10^6^	45.3 ± 1.3
140	30	5.5 × 10^6^	51.3 ± 1.5
140	10	5.1 × 10^6^	48.1 ± 1.3
140	60	7.2 × 10^6^	54.6 ± 2.2

### 4.4. Characterization of the Capacitive Acoustic Transducer

#### 4.4.1. Static Capacitance Measurement

**Figure 12:**
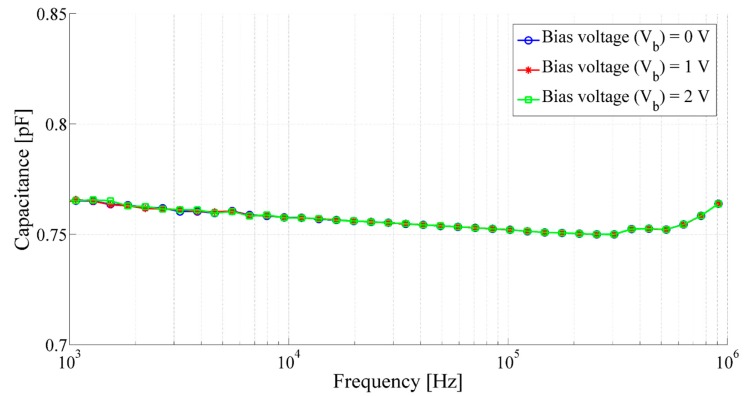
Frequency response of capacitance of the capacitive acoustic transducer with the membrane radius of 8.1 mm, backplate radius of 871 µm and air gap of 67.7 µm.

Static capacitance of the transducer with respect to the frequency (*f*) has been performed using an AC impedance spectrometer. It has been observed that the static capacitance of the transducer does not depends on the bias voltage and the state of charge of the capacitor. Figure 12 shows the frequency response of the capacitance of the transducer between 1 kHz to 1 MHz at different dc bias voltages.

The frequency response of the capacitive acoustic transducer shows almost no dependency for the applied bias voltage between 0 and 2 V. At frequencies between 1 kHz to 1 MHz the static capacitance of the printed capacitive transducer remain almost constant at 0.76 pF. Slight fluctuations of the capacitance at higher frequencies may occur due to the skin effect [45] or due to change in parasitic inductance [46] of the printed electrodes.

**Figure 13:**
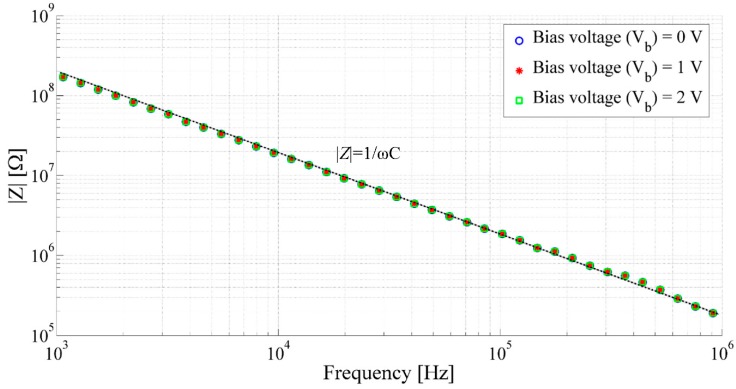
Frequency response of impedance of the capacitive acoustic transducer with the membrane radius of 8.1 mm, backplate radius of 871 µm and air gap of 67.7 µm.

In addition, Figure 13 shows the impedance behavior of the printed transducer with respect to frequency. It has been observed that the impedance |Z| decreases inversely with the frequency. This behavior is in agreement with the behavior of an ideal capacitor. Indeed the impedance Z of an ideal capacitor can be expressed as follows, where ω (=2π*f*) is the angular frequency and C represents the electrostatic capacitance of the capacitor:

(5) $$Z=\frac{1}{j\omega C}=\frac{1}{2\pi jfC}$$

**Figure 14:**
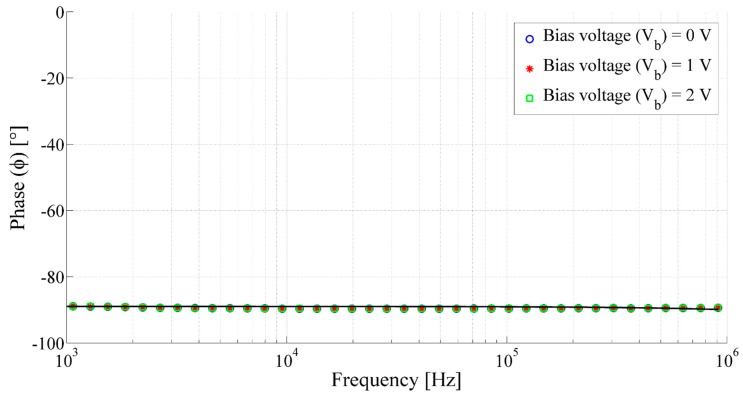
Frequency response of phase of the capacitive acoustic transducer with the membrane radius of 8.1 mm, backplate radius of 871 µm and air gap of 67.7 µm.

Moreover, the current and voltage of the transducer are not in phase with each other, as the voltage has a phase shift (ϕ) shift of around −90° with some fluctuations, as observed from the Figure 14, which is similar to that of an ideal capacitor. Therefore, the fabricated printed acoustic transducer has acted as a capacitor.

#### 4.4.2. Dynamic Characterization

**Figure 15:**
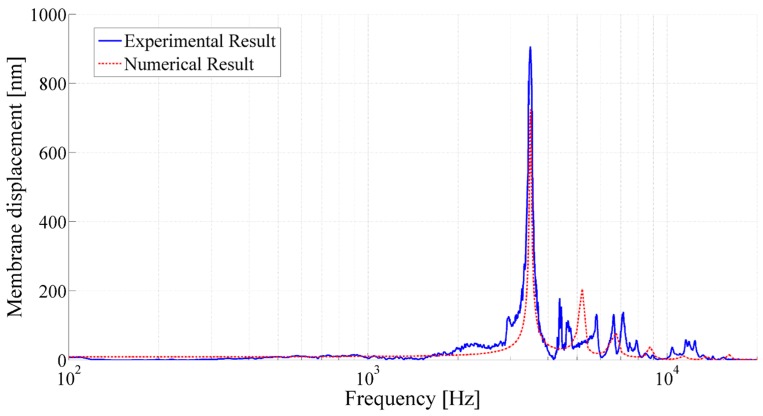
Frequency response of the printed acoustic transducer having membrane radius of 8.1 mm, backplate radius of 871 µm, cavity height of 3990 µm, air gap of 67.7 µm, membrane thickness of 23 µm and membrane tension of 48 N/m.

The capacitive acoustic transducers have also been characterized by LDV in single-point and scanning mode. It has been observed that the fabricated printed capacitive transducer exhibits strong sensitivity at the first resonance frequency. Experimental results were compared with the numerical simulation results. Figure 15 present the frequency response of the transducers under acoustic pressure. The maximum membrane displacement and calculated Q-factor of the resonators is listed in Table 4. The result shows good correlation between experimental and numerical measurements. However, the experimental results exhibit slightly different membrane displacement compared to the simulated results, and the simulated Q-factor of the transducers was higher than that of the experimental Q-factor as well. In addition, experimental measurements present some additional peaks or noise that could occur because of defects or non-linearity in the system. The defects might be generated during the assembly process due to the different tightness of the screws, which led to the non-linearity in membrane tension and also deforms the membrane in the system.

**Table 4:** Experimental results for the acoustic resonators.

Device Parameters						Numerical Results		Experimental Results	
*R_m_*	*R_B_*	*h_c_*	*h_g_*	*T_m_*	*t_m_*	*f*_10_	*Q_f_*	*f*_10_	*Q_f_*
mm	mm	mm	µm	N/m	µm	Hz		Hz	
8.1	0.87	3990	67.7	48	23	3489.2	61.6	3490	34

**Figure 16:**
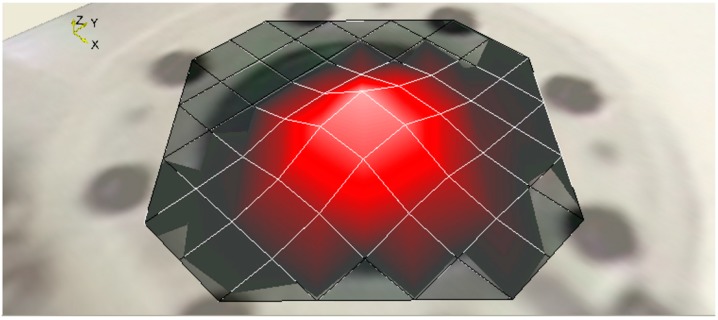
Modal shape of the membrane of the capacitive acoustic resonator, with membrane radius of 8.1 mm, backplate radius of 871 µm, cavity height of 3990 µm, air gap of 67.7 µm, membrane thickness of 23 µm and membrane tension of 48 N/m under acoustic pressure, at the resonance.

The use of scanning mode LDV measurements confirms that the first peak corresponds to the first resonance frequency of the membrane (Figure 16). The developed printed acoustic transducer consists of a much smaller backplate electrode compared to the membrane. Considering the membrane deformation at the center as uniform movement over the whole backplate surface, the relative capacitance variation (∆*C*) Scan also be calculated using Equation (1).

The printed acoustic transducer with a membrane radius of 8.1 mm, backplate radius of 871 µm, cavity height of 3990 µm, air gap of 67.7 µm, membrane thickness of 23 µm and membrane tension of 48 N/m, having static capacitance of 0.76 pF an approximate capacitance variation of 4.2 fF and Q-factor of 34 at its frequency of 3490 Hz have been computed, which exhibited close proximity to the numerical result exhibiting capacitance variation of 3.4 fF and Q-factor of 61.6.

**Figure 17:**
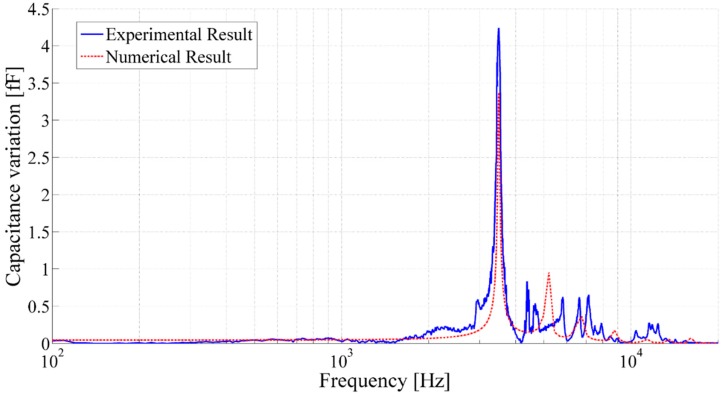
Capacitance variation with respect to the frequency of the printed acoustic transducer with the membrane radius of 8.1 mm, backplate radius of 871 µm, cavity height of 3990 µm, air gap of 67.7 µm, membrane thickness of 23 µm and membrane tension of 48 N/m.

Figure 17 presents the estimated capacitance variation based on the membrane displacement of the backplate electrode. The measurements have shown that the printed acoustic transducers performed as capacitive acoustic resonators that exhibit adequate sensitivity and selectivity at their first resonance.

## 5. Conclusions

A new approach where 3D printing and direct write 2D inkjet printing techniques are combined to fabricate capacitive acoustic transducers with selective sensitivity at their resonance has been successfully demonstrated,. In this work, we optimized the printing of conductive layers on 3D printed substrates to achieve proper conductivity without damaging the 3D structures, and also presented a technique to fabricate membranes with tension by a printing technique using thin organic films.

As the polyjet 3D printed materials have low heat deflection temperature, photonic sintering was used since it allows the sintering of printed silver layers on 3D printed substrates without damaging the 3D printed structures. The membrane was fabricated using a thin organic polymer film. Experiments revealed that the printing on the thin film has to be done on a pre-tensed film to fabricate the conductive membrane. When tension is applied after printing, the conductivity of the layer deteriorated due to appearance of cracks.

Static capacitance measurements showed that the fabricated printed transducer works as a pure capacitor. On the other hand, the dynamic measurements using LDV confirmed that the device provides high sensitivity and selectivity at its first resonance frequency, and exhibited good correlation with the numerical simulations. Therefore, the printed capacitive acoustic transducer performs as an acoustic resonator that exhibits adequate sensitivity and selectivity at its first resonance frequency with a Q-factor that was in agreement with the device requirements.

This kind of transducer can also be used for other interesting applications, such as navigation of drones, and autonomous systems or bio-mimetic development based on animal instincts [47], without the need to use filter circuits in applications where weight and power are critical, like in embedded systems for sound localization installed on very light autonomous micro-air vehicles [47]. The frequency of the transducer can be tuned by controlling the membrane tension or by changing the dimensions of the device depending on the application.

The fabrication approach that has been used during this work to develop capacitive acoustic transducers by combining 3D printing and 2D printing techniques, could be used profitably to fabricate a wide range of other 3D electronic devices and components as well, such as MEMS devices, strain sensors, pressure sensors, *etc*. In addition, other selective sintering techniques, such as microwave sintering, or infrared sintering techniques may be used to sinter the conductive layer on 3D printed structures.

## Supplementary Files

Supplementary File 1
