# Supplementary material for: Fabrication of Capacitive Acoustic Resonators Combining 3D Printing and 2D Inkjet Printing Techniques
Source: Sensors (Basel). 2015 Oct 14;15(10):26018–38. doi: 10.3390/s151026018 (PMC4634497; doi:10.3390/s151026018)
Supplement: Supplementary File 1 [file sensors-15-26018-s001.pdf]

OPEN ACCESS

sensors

ISSN 1424-8220

www.mdpi.com/journal/sensors

Supplementary Materials

Fabrication of Capacitive Acoustic Resonators Combining 3D Printing and 2D Inkjet Printing Techniques. *Sensors* 2015, *15*, 26018-26038

Rubaiyet Iftekharul Haque [1,*], Erick Ogam [2], Christophe Loussert [3], Patrick Benaben [1] and Xavier Boddaert [1,*]

[1] Centre Microélectronique de Provence (CMP), École Nationale Supérieure des Mines de Saint-Étienne, 13541 Gardanne, France; E-Mail: benaben@emse.fr

[2] Laboratoire de Mécanique et D'Acoustique UPR7051 CNRS, 31 Chemin Josep Aiguier, 13402 Marseille, France; E-Mail: ogam@lma.cnrs-mrs.fr

[3] TAGSYS RFID, 13600 La Ciotat, France; E-Mail: christophe.loussert@tagsysrfid.com

* Authors to whom correspondence should be addressed; E-Mails: haque@emse.fr (R.I.H.); boddaert@emse.fr (X.B.); Tel.: +33-4426-16761 (X.B.); Fax: +33-4426-16593 (X.B.).

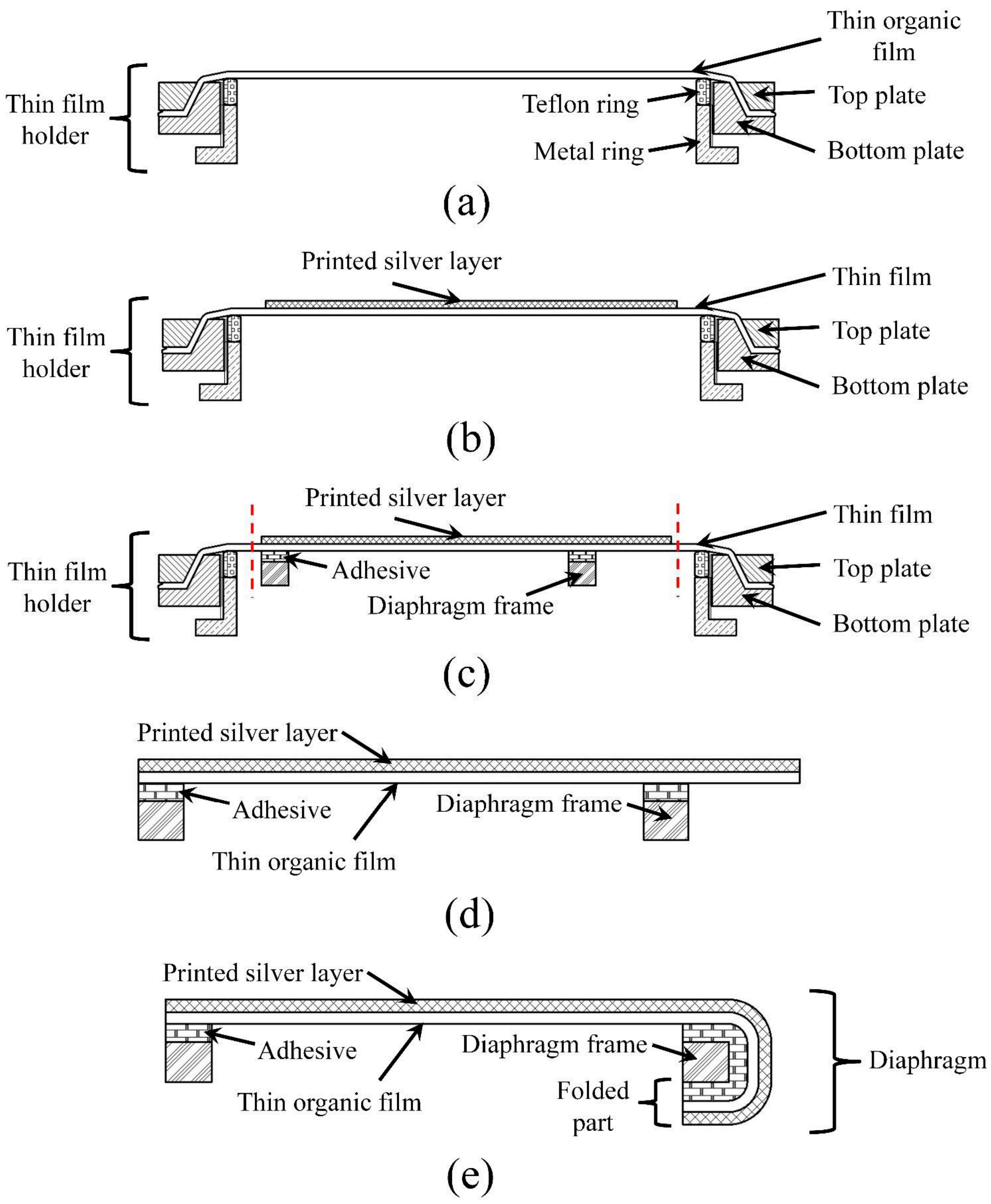

Figure S1. Schematic diagram of the membrane fabrication using thin film. Cross-sectional view of (**a**) thin film mounted on thin film holder; (**b**) printing and sintering of the conductive layer; (**c**) gluing the diaphragm frame to the thin film on the opposite side of the printed layer; (**d**) after separating of the diaphragm section from the film holder; (**e**) The final diaphragm after preparing the connection.

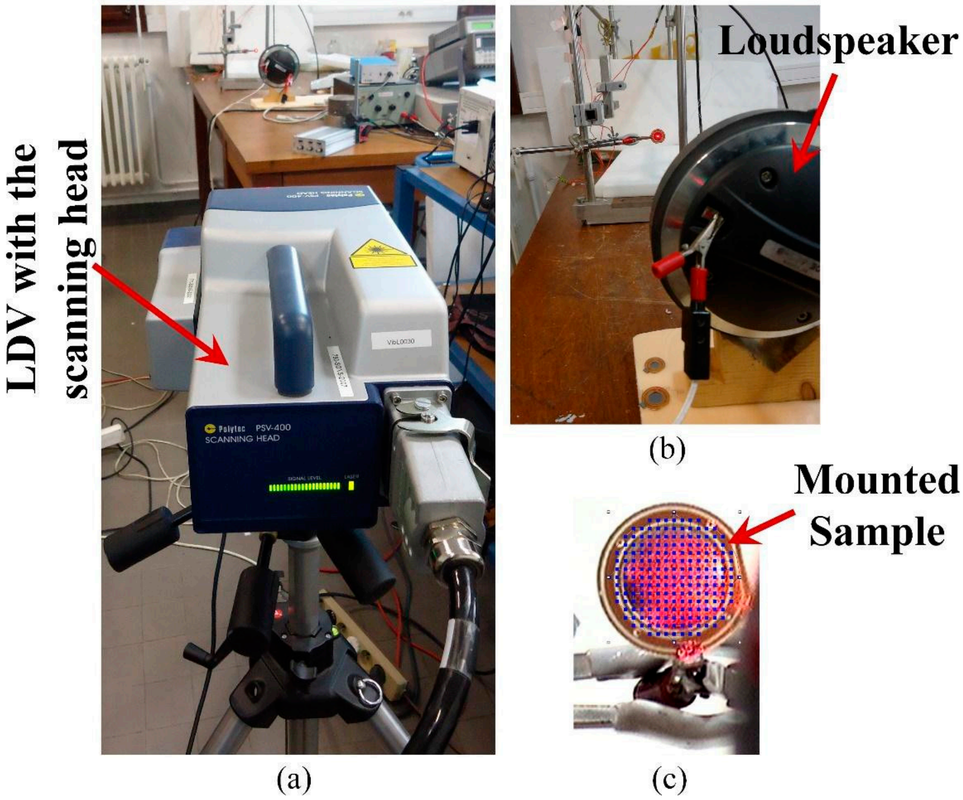

Figure S2. Setup for the dynamic response measurement of the membrane and transducer using LDV (**a**) full setup; (**b**) close view; and (**c**) selection of points for the scanning mode LDV measurement.

© 2015 by the authors; licensee MDPI, Basel, Switzerland. This article is an open access article distributed under the terms and conditions of the Creative Commons Attribution license (http://creativecommons.org/licenses/by/4.0/).
